# Supplementary material for: Causal interplay between lactose intolerance and gut microbiota: a combined bidirectional Mendelian randomization and in vivo validation study
Source: Front Nutr. 2026 Jun 1;13:1803337. doi: 10.3389/fnut.2026.1803337 (PMC13265576; doi:10.3389/fnut.2026.1803337)
Supplement: Supplementary file 1 [file Data_Sheet_1.zip › supplementary materials/Forward/forest plot/ebi-a-GCST90027648.finngen_R12_E4_LACTONAS.pdf]

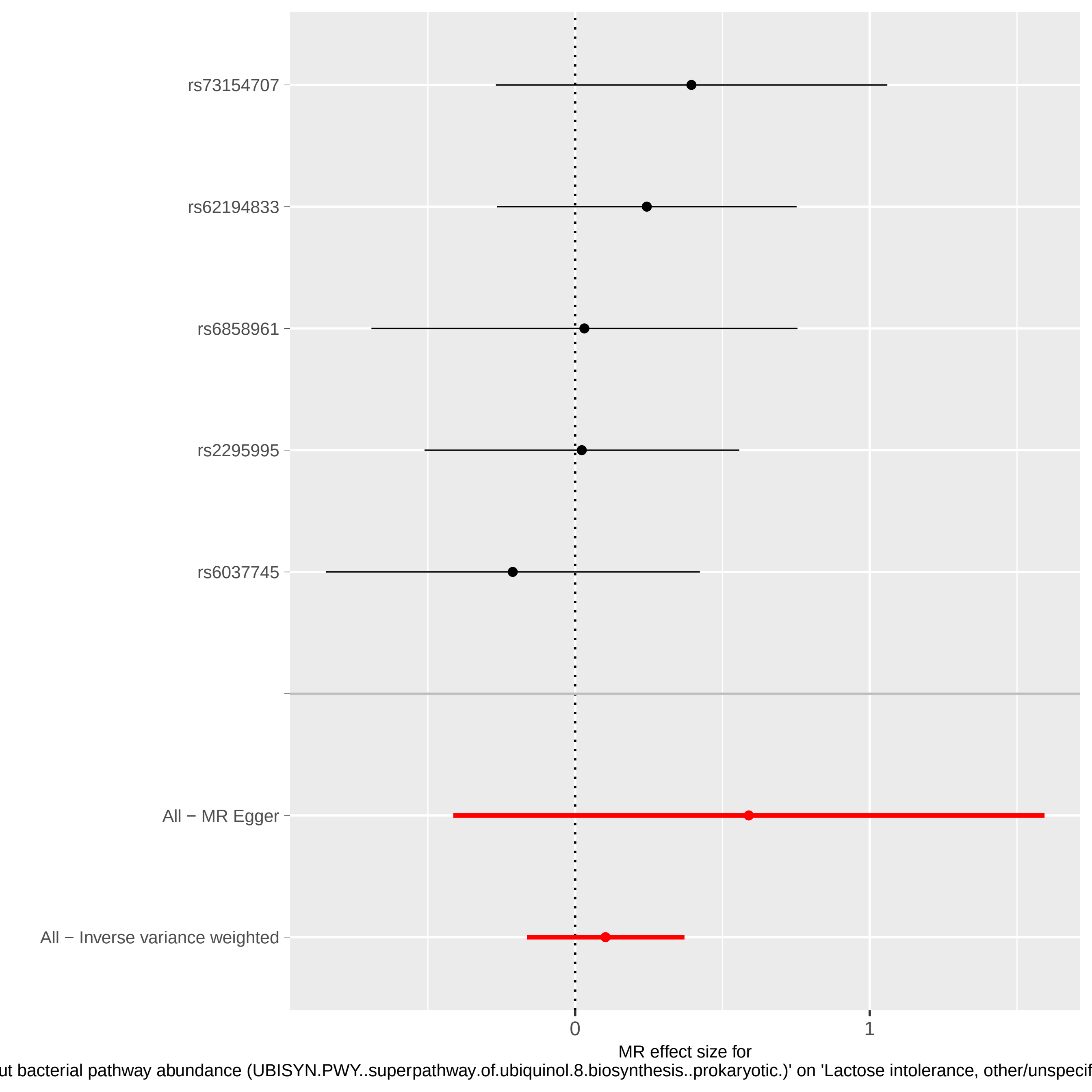
rs73154707
rs62194833
rs6858961
rs2295995
rs6037745
All − MR Egger
All − Inverse variance weighted
0
1
MR effect size for
ut bacterial pathway abundance (UBISYN.PWY..superpathway.of.ubiquinol.8.biosynthesis..prokaryotic.)' on 'Lactose intolerance, other/unspecif
